# Syphilinum

**Published:** 1891-07

**Authors:** Thomas Wildes

**Affiliations:** Kingston, Jamaica


### SYPHILINUM.

Thomas Wildes, M. D., Kingston, Jamaica.

This remedy, which I have acquired the habit of always using in the 1,000th potency, and which I invariably give once a day only, a dose every night on retiring, from my hands has cured nearly every one of the very many cases of Hunterian chancre that I have treated in the past fourteen years, unaided by any other medicine, the chancre or chancres growing larger for the first two weeks, and then gradually fading away from the margin toward the centre until they disappear in six, seven, or eight weeks, and are never followed by any secondary or tertiary symptoms. I prefer to get the cases during the first three days after the chancre appears.

In cases where the edges of the chancre assume the appearance of proud flesh in the third or fourth week, and become averted, jagged, and angry dark red, I substitute Lac-caninum$^{100,000}$, Swan, a dose every night for ten days or two weeks, and until the sore takes on a natural appearance, when I finish the cure with Syphilinum$^{1,000}$, Swan.

When, after healing, an indurated spot is left, I give Nitric-acid$^{30}$ four times a day until it disappears, having clinically learned its advantage over Silicea in such cases.

In Jamaica I have had abundant opportunity to verify the foregoing, as also I had in New York. Moreover, in New York I have lived to see my patients whom I had cured of syphilis marry and raise healthy children—in one instance three children—and never in any case could I, as a hereditarian, discern the slightest sign of syphilis in any of the children, either from their teeth or otherwise, nor in their mothers, either as uterine difficulties or otherwise. This is more than can be said in favor of any other method of treating syphilis that I have ever known of, or in favor of the treatment by other doctors in cases where it has fallen to my lot to treat the *after effects* in either the father, the mother, or the children of a man who has been syphilized.

I have in mind two men in this Island who, as I believe, I have cured of syphilis with Syphilinum, who have since married and have children, one each, and these children and their mothers are apparently well and strong. The *first* wife of one of these men, whom I treated for tertiary, had died with, from description, evident signs of syphilis while carrying her first child.

I cannot apply the children test to all of my cases, of course. Nor can I apply the larynx test to any of my cases, as that requires that fully twenty years shall elapse after the so-called cure was performed.

Where the cartilages of the throat are attacked, thus bringing the case to the notice of the laryngoscopist, it is usually eighteen or twenty years after the inception of syphilis, and, until the patient is questioned, he has forgotten his former "difficulty with a woman." Therefore, I do not consider any of my cases, dating back only fourteen years, as absolute cures as yet.

It is also early for me to apply the crucial test of TIME to my cases of cures of primary, secondary, or tertiary syphilis in Jamaica, and until later I am not prepared to state as a fact, though I believe it to be true, *that there is no longer any trace of syphilis in the blood of those I have cured in this Island.*

Syphilinum has cured more headaches among my patients than has any other remedy. Its chief headaches are: linear, from or near one eye backward; lateral headaches, frontal headaches, headaches often from temple to temple, or deep into the brain from vertex, or both; headaches as from pressure on vertex; violent headache in either temple, extending into or from the eye, relieved by warmth; violent pain in the bones of the head or face; headaches all aggravated by the heat of the sun, after effects of sunstroke, are cured by Syphilinum. Headaches are usually accompanied by great restlessness, sleeplessness, and general nervous erethism. Syphilinum cures the headaches of Kali-bichrom., Spigelia, Sanguinaria, Silicea, Aranea-diadema, Agaricus, Stannum, Mezereum, Lac-felinum, and many others. It cures headaches through temples, thence vertically, like an inverted letter T, accompanied with eye symptoms of incipient tumor of the optic chiasm and stale, mouldy, fetid breath. It cures ptosis. It relieves paralysis of the superior oblique. It cures aphasia in its various forms, with its full complement of concomitants, including, as in one case cured here, facial paralysis (seventh pair), left side, ptosis and sudden paralysis of optic nerve of left eye, causing many weeks of blindness; partial paralysis of tongue, which was protruded, crooked, sluggish, heaviness of speech, complete hemiplegia for first thirty-six hours, afterward partial for several weeks. I took the case after an allopath had treated him for six weeks, and thereafter he progressed steadily to a cure.

Very many such cases occur here, the persons believing themselves to be "*moon-struck.*" The allopaths frankly avow their helplessness in such cases. I have cured many—all who came.

One case, with facial paralysis, right side, thick speech, hemicrania, jactitation of right eye and lid, was cured in five weeks, after the allopaths had given her up, in a woman of forty. Other cases, and many, I have cured in three weeks. One old gentleman of seventy-six, a retired minister, with thick speech, difficulty of finding his words, and with debility and other remnants of aphasia, I cured in three weeks, so that he was strong and well and talked as straight as any one, after he had been tinkered at and given up as a hopeless case by allopaths for about one year and a half, and had also tried electricity unavailingly. That was three years ago this month, and the old gentleman is still alive and hearty.

It cures periosteal pains anywhere in the body.

It cures the chronic congested spots on the eye, more often on the temporal side, and usually about one to three lines behind the cornea, of a dark red color, apparently imbedded in the sclera, which are always indicative of syphilis.

In cases of acute attacks resulting from tertiary syphilis, such as disease of the cartilages of the throat, or of the periosteum, the action of Syphilinum is often violent at first, and brings on decided aggravations. So also will it aggravate at first the acute pharyngitis of secondary syphilis, or the acute nervous erethism, or acute syphilitic paralysis of the tertiary stage, but it hastens and promotes the cure of all.

Syphilinum has been of great help in my cases of catarrhal and nerve deafness, where a marked cachexia exists. Two such cases are now under my care and markedly improving. One, a grown man, is also gaining strength rapidly, after repeated slight pulmonary hemorrhages for four years past.

It cures itching in the nostril. Attacks of fluent coryza. Dark purple lines between the alæ nasi and the cheeks.

Always curative in itching, scabby, eczematous eruptions when they appear on the face or breast, singly, or in clusters, and looking like herpes.

It cures pain and pressure behind the sternum.

It first aggravates, and afterward is curative, in cases of so-called *heartburn,* with pain and rawness extending from stomach to throat-pit, and often accompanied by cough.

It promptly relieves, and eventually hastens the cure of violent attacks of dyspnœa, with wheezing and rattling of mucus, coming on from one to four A. M., whether from emphysema, capillary bronchitis, or simple asthma proper, and is always a valuable adjunct remedy in such cases.

It cures dyspepsia. Daily vomiting for weeks or months, due to erosion from superficial ulceration of lining of viscus. I claim this erosion or ulceration to be due to herpetic rash of syphilitic origin.

It cures dyspepsia, flatulence, belching of wind. Cures all *nervous dyspepsia* when not originating in pressure at base of brain, in which case *Psorinum* is more often the remedy, especially in the absence of syphilitic cachexia.

It is the best temperance advocate on earth, and cures tendency to heavy drinking; all habitual drunkards being syphilitics.

Its action on the liver is beneficial and lasting. In chronic constipation, with fetid breath, earthy complexion, gaunt appearance, it produces desire for stool every morning, which stools are yellow and easily passed, bringing great relief.

It restores appetite when capricious or scant, or absent, during melancholy moods.

It cures yellow leucorrhœa of offensive odor, either watery or not, and so profuse that it daily soaks through the napkins and runs down to the heels of the stockings, if the woman is much on her feet—at least six cases in my practice since 1877–8.

It cures the profuse leucorrhœa so often found in sickly, nervous children of five to ten years of age.

I believe that it often prevents the formation of ovarian tumors and cures congestion and inflammation of the ovaries and fallopian tubes, and I know that it cures inflammations and indurations of the spermatic cord.

It is an indispensable remedy in many cases of uterine and ovarian diseases, especially in married women, and particularly if accompanied with pronounced nervous disorders. It reaches the cause; for many a married woman carries with her to her (often early) grave, either oophoritis, salpingitis, metritis, ulcerations, congestions, or hydatids, the result of latent syphilis or gonorrhœa in her husband, transmitted to her and to her children.

It quickly allays the pain of rheumatism of shoulder joint, or at insertion of deltoid, which is aggravated by attempts to raise the arm laterally, and in a few days the mobility of the joint is greatly increased. It hastens the cure of the case.

It cures rheumatism where the muscles are caked in hard knots or lumps. Also cases of excruciating arthritis, where the swelling, heat, and redness are intense.

Occasional doses of Syphilinum or Psorium are indispensable in obstinate cases of cholera infantum.

Syphilinum often aggravates the acute ophthalmia neonatorum. I have always found it better to cure the ophthalmia first, and then drive the syphilis out afterward.

It vies with Sulphur in its wonderful power to produce a quiet, refreshing sleep.

It has cured for me many cases of epilepsy.

It causes and cures a contracted and painful feeling in the soles of the feet, as if the tendons were too short.

In 1876, I cured with Syphilinum$^{m}$, Swan, a bookkeeper in New York who had suffered for many months with a piercing, pressing, excruciating headache over the right eye and extending deep into the brain. It was so severe that he was losing his continuity of thought and his memory.

He made repeated mistakes in figures, and often made out and sent bills twice, on consecutive days, unnecessarily, to the same persons.

He was in danger of losing his situation. Under this remedy, a dose every night, his headache entirely disappeared in ten days, and his mental faculties were fully restored.

In six weeks his whole eyebrow on that side broke out in a scabby, yellow, syphilitic eczema, with a red, angry, and oozing base, extending under the arch to the lid, also a finger's width upward on the forehead, and from canthus to canthus, and down on and partly across the nose—the whole being not only unsightly but also very tedious and most difficult to heal, because of my folly in changing my medicine too often. I should have stuck to Syphilinum. When the rash appeared and I questioned him, he then acknowledged what he had denied before, viz.: that he had had syphilis a few years before, but claimed that it had been *cured*. He is now manager of an institution that is known the world over.

In 1878 there came to me an operator in the employ of the Western Union Telegraph Company, who had known me in a previous boarding-house. He had suffered from chancre for nearly six weeks, and the only results of the repeated nitric-acid-burning treatment of his former physician was that the chancre had eaten its way freely from the frænum up the left side of the glans penis, thence backward, involving the foreskin through and through, thence backward, involving even the deep tissues of the cuticle almost to the root of the penis, and half a finger breadth in width. I told him that his blood was already involved, and that I could not prevent secondary and tertiary symptoms coming. They did come, and with a vengeance, so soon as he began to take Syphilinum$^{m}$, Swan. Abscesses and ulcers formed in his throat, and his face, scalp, body, and extremities broke out in a violent syphilitic rash, as thick as measles, angry-red, each papule developing a scab which would scale off to give place to another. For several weeks he was a holy show! The chancre gradually healed, and finally the syphilitic rash followed the throat trouble and disappeared. Then came headaches and *plaques mucosa*, the latter covering the vermilion border of both lips, which were swelled, and lying in patches in the mucous lining of his mouth, and affecting the edges of his tongue. His anus also began to show signs of rawness, fissures, etc. He took the Syphilinum every night at bedtime, and in about five months I pronounced him cured. He afterward married, and five or six years ago I had the pleasure of seeing his then three maiden efforts in the shape of matrimonial bonds, two of them with coupons on, and I could discover no evidence of syphilis in either one of those three children, and yet I was looking to find it.

Next to our old Mayor, Fernando Wood, who had "an eye for the public good," I believe that I have the keenest eye for detecting syphilis of any man in either school of medicine.

In 1879, a lady came to me for a dreadful ozœna. She also had curvature of the spine, and a congestive trouble of the right ovary which was so pronounced that I sent her to a specialist to be examined for ovarian tumor. She was an extremely bright and intelligent young woman of about twenty-six or twenty-seven years, but from a child had always been very delicate. Her mother, whom I know but little, was also always a very delicate woman with some eczema trouble, but a woman of great nervous energy. Syphilinum cured the ozœna, and helped her health, but, *miserere*, it drove out a *saddle* to which the Sepia saddle is not even a shadow! It consisted of a furious, inflammatory mass of syphilitic sores, scabs, and eczema, red and angry, with a fiery base, extending from one malar prominence to the other, across the nose, up to the eyelids, and on the forehead, chiefly over the frontal sinuses. It was a horrible disfigurement for a young woman, and I confess to being eighteen months removing the rash and with it the hideous picture that her face presented. Her ozœna never returned, she is still living and enjoys fairly good health. I learn that her brother's child, five years old, is now being treated for obstinate catarrh. He will soon get Syphilinum$^{m}$, Swan.

In 1883, I cured a man who lived on Hudson Street, New York, of syphilitic destruction of hard and soft palates. He was sixty years old, and admitted having had syphilis many years before. There was an open hole from the floor of the nares to the roof of the mouth about one inch long and a half inch wide. The soft palate was gone, and the pillars were going. He could not swallow liquids except he held his nose to prevent escape. After many weeks of the steady use of Syphilinum, the destructive process ceased, and the parts healed in their then denuded state. It was my first case of the kind, and so soon as the fresh surfaces had healed, I gave Aurum-mur.$^{30}$, four times a day for several weeks, for fear that the bones would go bad again. My later experiences have shown that Aurum is never necessary.

Recently I have cured of the same thing, same parts involved, and same destruction of tissue, a boy of ten, whose brother is in the Post Office here. I can get no history of syphilis, but Syphilinum cured him.

I now have on hand a precisely similar case in an old black woman who is recovering. She fights shy of history. I give her Syphilinum.

[TO BE CONTINUED.]

